# Antioxidant Bio-Based and Biodegradable Polymer Films for Sustainable Food Packaging

**DOI:** 10.3390/ma19091797

**Published:** 2026-04-28

**Authors:** Maria Letícia de Sousa Gomes, Francisco Xavier Nobre, Lucas de Souza Falcão, Mariana Agostini de Moraes, Patrícia Melchionna Albuquerque

**Affiliations:** 1 Multicentric Postgraduate Programme in Biochemistry and Molecular Biology, School of Health Sciences, Amazonas State University, Av. Carvalho Leal, 1777, Manaus 69065-020, Brazil; m.leticiarep@hotmail.com (M.L.d.S.G.); francisco.nobre@ifam.edu.br (F.X.N.); 2 Laboratory of Chemistry Applied to Technology (GP-QAT), School of Technology, Amazonas State University, Av. Darcy Vargas, 1200, Manaus 69050-020, Brazil; lucas.sfalcao@hotmail.com; 3 Group of Energy Resources and Nanomaterials (GREEN), Department of Chemistry, Environment, and Food (DQA), Federal Institute of Education, Science and Technology of Amazonas, Manaus 69020-120, Brazil; 4 Department of Materials Engineering and Bioprocesses, School of Chemical Engineering, Universidade Estadual de Campinas, Av. Albert Einstein 500, Campinas 13083-852, Brazil; agostini@unicamp.br; 5 Postgraduate Programme in Biotechnology and Amazon Natural Resources, School of Health Sciences, Amazonas State University, Av. Carvalho Leal, 1777, Manaus 69065-020, Brazil

**Keywords:** biopolymer films, antioxidant packaging, polymer materials, bio-based polymers, diffusion-controlled release, active food packaging, bibliometric review

## Abstract

Antioxidant biopolymeric films (ABFs) have emerged as promising bio-based and biodegradable polymer materials for sustainable food packaging, combining environmental sustainability with functional performance. This study identifies convergent design principles governing ABFs through a systematic mapping of research published between 2015 and 2025, organized into thematic discussions covering global trends, material strategies, processing technologies, and structure–property relationships. The analysis reveals a clear transition from biodegradable substitution materials toward performance-driven polymer systems engineered to modulate mass transport phenomena. Polysaccharide- and protein-based matrices dominate current developments due to their chemical functionality and compatibility with natural bioactive compounds; however, their inherent hydrophilicity introduces trade-offs between barrier resistance and controlled release. Recent advances increasingly employ blends, composites, and multilayer architectures to decouple mechanical stability from antioxidant migration. Processing technologies, including casting, extrusion, and multilayer assembly, are shown to play a decisive role in defining diffusion pathways and release kinetics. The findings demonstrate that the effectiveness of ABFs depends primarily on polymer–bioactive interactions and structure–property relationships rather than additive concentration alone. Future progress toward industrial implementation requires scalable fabrication strategies and predictive processing–structure–performance frameworks aligned with circular economy principles. This perspective positions ABFs as functional bio-based polymer systems capable of synchronizing antioxidant release with food oxidation kinetics, contributing to sustainable food packaging solutions.

## 1. Introduction

Environmental impacts associated with industrial activities—including food production, textile processing, plastics manufacturing, energy generation, and biofuel production—have intensified global concerns regarding sustainable technologies to mitigate long-term risks to ecosystems and public health [1]. In this context, the Sustainable Development Goals (SDGs), adopted by United Nations member states as part of the 2030 Agenda, provide an international framework to guide strategies aimed at promoting sustainable production systems and reducing environmental pressures [2,3]. These goals encourage the reduction in greenhouse gas emissions, the proper management of persistent pollutants, the conservation of natural resources and ecosystems, and the development of industrial processes that minimize environmental impacts while supporting economic and social development [4].

Among the pollutants considered particularly harmful to ecological balance and human health are toxic metals [5], organic solvents [6], and persistent organic compounds (POCs), including textile dyes [7], pesticides [8,9], antibiotics [10], hormones [11], and plastic materials [12]. Their widespread environmental dispersion, particularly through aquatic systems, represents a major concern as it directly affects water quality and ecosystem stability. The persistence of synthetic polymers in the environment has therefore stimulated the search for alternative materials capable of reducing long-term ecological impacts.

Microplastics illustrate the scale of this challenge. Pegado et al. [13] reported the presence of microplastic particles in the digestive tracts of 189 fish specimens from 46 species collected along the northern coast of Brazil, covering an area of approximately 488,000 km^2^. Using Fourier transform infrared spectroscopy (FTIR), the authors identified 288 plastic particles classified as pellets, sheets, fragments, and fibers, with sizes ranging from 0.38 to 4.16 mm. Similarly, Rojas et al. [14] documented the occurrence of microplastics in fish from the Peruvian Amazon, identifying 2337 particles accumulated in gills and digestive tracts of specimens from local markets in Iquitos. These findings highlight the persistence and mobility of synthetic polymer debris within aquatic ecosystems and reinforce the need for more sustainable material alternatives.

In response to these challenges, increasing attention has been directed toward the development of bio-based and biodegradable polymer materials for food packaging applications. Among these alternatives, biopolymeric films have emerged as promising candidates for sustainable food packaging materials, as they combine environmental compatibility with functional performance. These materials are typically produced from polysaccharides or proteins derived from renewable sources and may exhibit biodegradability while maintaining adequate physicochemical properties for packaging applications [15].

Biopolymeric films are generally composed of a polymeric matrix, a solvent system, and functional additives, and they can be processed using techniques such as solution casting or extrusion. When applied directly to food products, coating strategies including dipping, spraying, or spreading may be employed [16,17,18,19].

From a materials science standpoint, antioxidant biopolymeric films represent functional polymer systems in which transport phenomena, polymer–additive interactions, and processing conditions collectively determine antioxidant performance. In food systems, these materials may function either as standalone packaging structures or as edible coatings capable of extending the shelf life of food products. Beyond food preservation, biopolymeric films have also been investigated for pharmaceutical and cosmetic applications due to their compatibility with bioactive compounds [20,21,22].

To enhance packaging functionality, these films can be engineered as active materials through the incorporation of additives with antioxidant or antimicrobial activity [23]. Oxidation reactions are a major cause of quality deterioration in food systems, affecting sensory attributes, nutritional stability, and safety. Consequently, the incorporation of antioxidant compounds—including enzymes, phenolic molecules, and organic acids—has been explored as a strategy to delay oxidative degradation processes [24].

However, the incorporation of bioactive molecules into polymeric matrices introduces important challenges related to polymer–additive interactions and controlled release behavior. These interactions may significantly influence the physicochemical properties of the film, including its diffusion characteristics and release kinetics [25]. The complexity increases further when considering industrial scalability and commercial applications, where materials must maintain performance during storage and processing conditions while effectively delaying deterioration in products such as vegetables, meats, fish, and dairy products [26].

Understanding how these materials have evolved and which strategies are driving their development requires a systematic evaluation of the scientific literature. Bibliometric analysis provides a useful approach for identifying global research trends, collaboration networks, and emerging technological directions. This method allows rapid and structured mapping of scientific production using large publication databases [27,28,29].

Bibliometric methods enable the quantitative exploration of scientific knowledge structures, including relationships between authors, institutions, and thematic domains. Such analyses typically employ specialized tools, including VOSviewer v. 1.6.20 and Origin 2024b combined with major scientific databases such as Web of Science and Scopus [30].

For instance, Zhu et al. [31] conducted a bibliometric study of the journal Advanced Healthcare Materials, analyzing publications between 2012 and 2019 using data retrieved from the Web of Science and visualized with VOSviewer v. 1.6.20. Their work evaluated annual publication output, citation patterns, contributing countries, and author impact metrics, identifying major research topics through keyword co-occurrence and providing critical perspectives on the clinical translation of engineered biomaterials. Similarly, Muktiarni et al. [32] investigated advances in thermoplastic starch research through a bibliometric study based on 213 documents retrieved from Scopus. Their analysis identified major research hubs and highlighted biodegradation processes and plastic waste management as key research directions.

Despite the rapid growth of research on antioxidant biopolymeric films for packaging applications, a comprehensive bibliometric evaluation focused specifically on this topic remains limited. Such an assessment is essential for identifying global research trends, technological priorities, and emerging directions in the development of functional polymer systems for sustainable packaging. From a materials science perspective, these systems should be understood as functional polymer materials in which transport phenomena, polymer–bioactive interactions, and processing-induced microstructure collectively determine antioxidant performance. These materials thus represent a strategic class of sustainable food packaging materials capable of integrating polymer science, active packaging technologies, and circular bioeconomy principles.

Therefore, the present study performs a bibliometric analysis of the scientific literature related to antioxidant biopolymeric films, focusing on publications indexed in the Scopus database between 2015 and 2025. All data extraction and organization steps were performed systematically to ensure consistency and minimize subjective bias in the interpretation of bibliometric and thematic results. This bibliometric mapping serves as a structural guide to identify the predominant research clusters, which are subsequently expanded into a thematic discussion. The analysis examines publication trends, journals, subject areas, and keyword patterns, enabling a comprehensive evaluation of the geographic evolution, research output, and the core technological features of bio-based antioxidant films.

This review is built upon the thesis that the field of ABFs has transitioned from simple material substitution toward performance-driven polymer engineering. To provide a clear understanding of the field’s evolution and potential, this review is structured as follows: Section 3 addresses the global research landscape; Section 4 identifies hot research topics; followed by a deep dive into materials and design strategies (Section 5), structure–property relationships (Section 6), and application fields (Section 7). Finally, research gaps and future directions are identified in Section 8.

## 2. Materials and Methods

The search strategy was iteratively refined through preliminary tests to balance sensitivity and specificity, ensuring the inclusion of relevant studies while minimizing irrelevant records. The final search query applied in the Scopus database was: **TITLE-ABS-KEY (“biodegradable film*” OR “biopolymer film*” OR “edible film*” OR “active packaging”) AND TITLE-ABS-KEY (“antioxidant*” OR “bioactive compound*” OR “natural extract*”)**, with no restriction on document type or publication year at the initial stage. The complete query is provided to ensure full reproducibility of the bibliometric dataset. Although this study incorporates elements of systematic searching, it does not strictly follow a PRISMA protocol, as the primary objective is not a systematic review of clinical or experimental outcomes, but rather a hybrid bibliometric–thematic analysis aimed at mapping research trends and guiding in-depth discussion.

The search was restricted to original research and review articles published in English. Initially, a broad search was performed without a time limit; however, to assess the state of the art, the analysis was refined to include only publications from the past ten years (2015–2025). The study was conducted in September 2025. Metadata for the bibliometric mapping (Section 1, Section 2, Section 3 and Section 4) were exported in RIS format, including titles, abstracts, authors, year, citation count, source, affiliations, language, and both author and indexed keywords. The screening process followed a structured approach involving (i) duplicate removal, (ii) title and abstract screening, and (iii) full-text assessment when required, ensuring thematic consistency with the scope of antioxidant biodegradable films.

The data collected from Scopus were systematically organized and tabulated in an Excel spreadsheet to rank the top 10 journals, thematic areas, countries, institutions, and authors, along with their respective percentage contributions. To visualize publication and citation trends, graphs were generated using Origin 2024b. Additionally, VOSviewer (version 1.6.20) was employed to analyze keyword co-occurrence in original research and review articles containing the terms biopolymer* AND antioxidant* AND film* from 2015 to 2025.

A representative subset of 173 studies was strategically selected to provide mechanistic and application-oriented insights. This approach is consistent with hybrid bibliometric–thematic reviews, where quantitative mapping is used to guide targeted qualitative analysis. Selection criteria included citation impact, recency, relevance to dominant keyword clusters, and the ability to exemplify matrix–bioactive interactions. The keyword co-occurrence analysis was specifically employed as a content-selection framework to bridge the bibliometric mapping with the thematic discussion. By identifying the most influential clusters, such as biopolymer matrices, processing technologies, and bioactive functionalization, this study transitions from a quantitative macro-scale analysis to a qualitative descriptive approach. Consequently, the subsequent Section 5, Section 6, Section 7 and Section 8 provide an integrated discussion of the current state of the art, focusing on the materials and strategies identified as trends in the bibliometric dataset.

**Figure 1:**
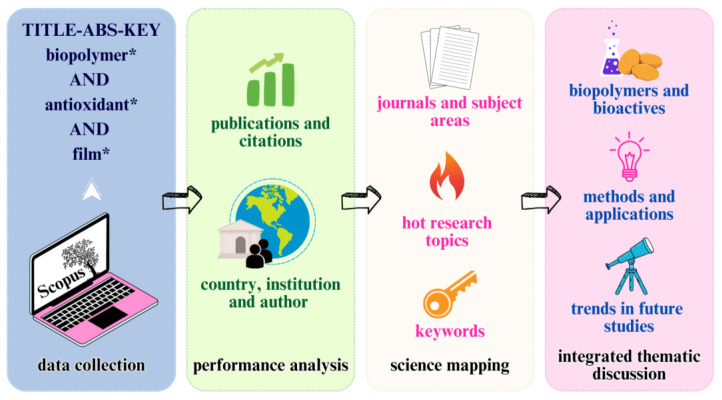
Methodological workflow: from bibliometric data acquisition and science mapping to qualitative thematic synthesis. The co-occurrence analysis (Science Mapping) serves as a framework for identifying core research trends guiding the integrated discussion on biopolymers, bioactive compounds, and emerging applications. * Truncation symbol.

Figure 1 illustrates the methodological workflow adopted in this study, centered on data acquisition and scientific mapping. The keyword co-occurrence analysis performed in VOSviewer v. 1.6.20 served as a thematic compass, identifying core clusters and emerging trends that structured the subsequent qualitative discussion. The bibliometric mapping not only identifies research trends but also supports the thematic organization of the discussion, allowing key materials, bioactive compounds, and processing strategies to be examined within the context of their scientific prominence and evolution.

## 3. Global Research Landscape of Antioxidant Biopolymeric Films

### 3.1. Publication Trends in Antioxidant Biopolymeric Films Research

The data presented in Table 1 indicates that, among the analyzed keywords, biopolymer* yielded the lowest number of scientific publications, totaling 62,515 articles over the entire search period. When restricted to the past ten years, this number decreased to 38,472 research articles, corresponding to 61.54% of the total. Similarly, the search term antioxidant* retrieved 595,177 articles, of which 398,384 (66.94%) were published in the last decade. These results suggest that both biopolymers and antioxidants represent consolidated research domains, whereas their combined application has intensified only recently.

The term film* produced the highest number of publications, with 1,290,826 articles. After applying the ten-year filter, 537,813 publications (41.66%) remained, indicating that film research predates the current interest in functionalization. This distinction reveals a shift in research focus from structural packaging materials toward functional polymer systems designed to regulate chemical and mass transport processes.

When analyzing keyword combinations, first as pairs and then as a combined search, the combination antioxidant* AND film* resulted in 8941 publications, of which 7687 (85.97%) were published between 2015 and 17 September 2025. The predominance of recent studies indicates that antioxidant incorporation represents a functional upgrade of traditional film materials, transforming them from inert barriers into active interfaces designed to modulate oxidation reactions.

A similar pattern was observed when all three keywords (biopolymer* AND antioxidant* AND film*) were combined, significantly narrowing the search scope; 97.64% of the resulting studies were published within the last decade. This trend indicates that antioxidant functionality is not an intrinsic feature of early biodegradable materials but rather a second-generation design strategy in which polymer matrices are engineered to provide chemical stabilization functions.

**Figure 2:**
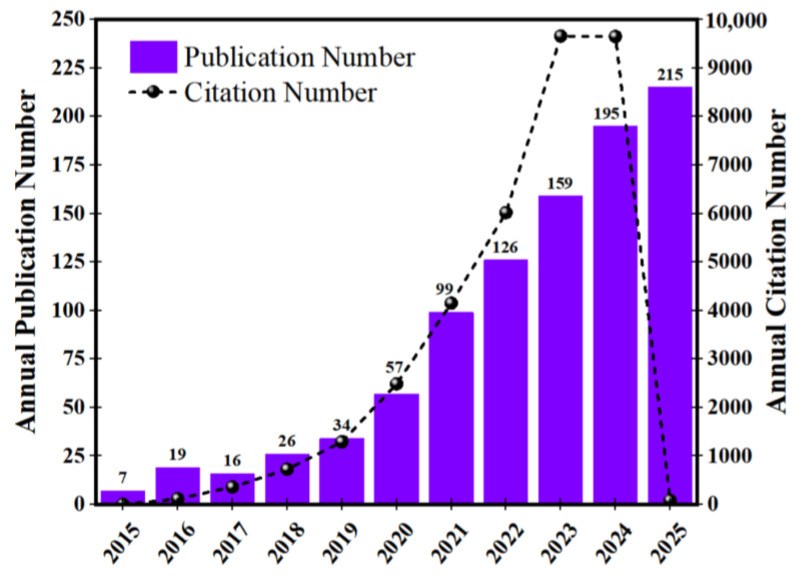
Temporal evolution of publications and citations demonstrating the transition from biodegradable materials research to functional polymer engineering in antioxidant films, indicating technological maturation rather than the emergence of a new research topic.

Figure 2 presents the annual number of publications and citations associated with ABFs. The publication output exhibited an average annual growth rate of approximately 40.84%, while the mean number of citations per article over the ten-year period was 33.83. Beyond bibliometric expansion, this trend reflects the consolidation of functional packaging as a materials engineering field in which polymer structure is tailored to control mass transport phenomena and oxidative stability. The simultaneous growth in publication volume and citation frequency suggests convergence toward performance-driven polymer design rather than simple environmental substitution.

The most cited studies are based on polysaccharide matrices, highlighting their relevance as model systems for investigating diffusion-controlled stabilization mechanisms in polymer networks. The leading article, with 545 citations, entitled “Development of edible films and coatings from alginates and carrageenans,” was published in Carbohydrate Polymers by Tavassoli-Kafrani, Shekarchizadeh, and Masoudpour-Behabadi [33] in 2016. The second most cited work, “Edible films and coatings in seafood preservation: A review,” published in 2018 by Dehghani, Hosseini, and Regenstein [18] in Food and Bioprocess Technology, has accumulated 483 citations. Together, these studies emphasize the role of polysaccharide matrices in elucidating the relationship between polymer structure, molecular diffusion, and oxidative stabilization.

These bibliometric patterns provide quantitative evidence supporting the central thesis of this review, namely that the field of antioxidant biopolymeric films has evolved from environmentally driven material substitution toward performance-oriented polymer engineering. The sharp increase in combined keyword usage and citation density reflects a shift toward systems designed to actively regulate oxidation processes rather than merely replace conventional plastics.

### 3.2. Scientific Structure and Collaboration Patterns

**Figure 3:**
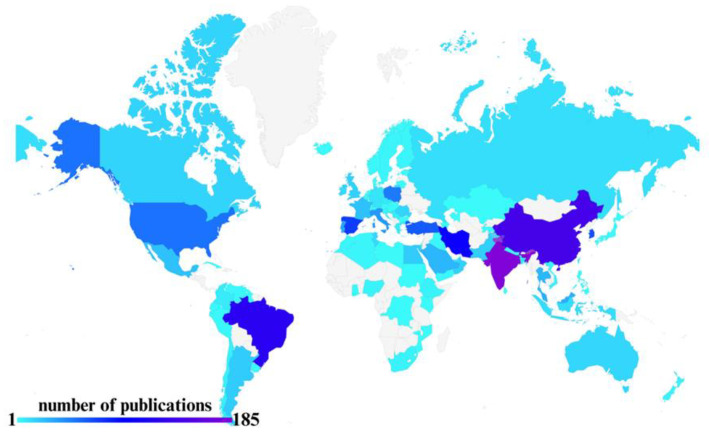
Global research hubs associated with development of functional polymer packaging systems.

Figure 3 presents the distribution of scientific publications by country of origin, while Table 2 ranks countries, institutions, and authors according to publication output. This overview contextualizes research efforts and highlights leading contributors, providing insight into where technological developments in materials design and applications are most active, as well as identifying the researchers who drive these developments in the mapped regions. The geographical distribution reveals a concentration of research activity in regions with strong integration between food science and polymer engineering, indicating that the development of ABFs is primarily driven by application-oriented materials design rather than by isolated advances in fundamental polymer synthesis.

Although the ranking was intended to include the top ten institutions, twelve institutions appear due to ties in publication numbers, as no additional tiebreaking criteria were applied. Together, these institutions contributed 246 articles, corresponding to approximately 25.81% of the retrieved publications. The leading institutions are located in South Korea, Brazil, and Iran, highlighting the presence of consolidated research hubs dedicated to functional packaging materials. These hubs coincide with regions traditionally active in food hydrocolloids and polymer interface science, reinforcing the application-driven nature of the field.

The predominance of certain countries in this field may be associated with the availability of biomass resources, investment in sustainable materials research, and regulatory pressure to replace synthetic packaging, highlighting both scientific and socio-economic drivers of research activity. These patterns also suggest potential opportunities for international collaboration, particularly between regions with strong materials science expertise and those rich in biodiversity and natural bioactive sources.

Despite the large number of contributing researchers, a small group of authors accounts for a substantial share of the scientific output, with Jong-Whan Rhim emerging as the most productive contributor. Within the dataset analyzed (see Table 2), he presents the highest h-index (19), with 28 publications and 1806 citations. It is important to note that these h-index values refer exclusively to the 953 documents retrieved through the search strategy and do not represent the authors’ overall bibliometric indicators. For instance, at the time of data collection, Dr. Rhim’s general h-index was 110, whereas Dr. Jamróz’s was 31.

Iran and Brazil occupy the second position, each with 24 publications (2.52% of total output), accumulating 1240 and 853 citations, respectively. The prominence of institutions such as the University of São Paulo reflects the existence of sustained research infrastructures supporting polymer-based materials development, which contributes to the consolidation of regional expertise and collaborative networks within this field.

### 3.3. Knowledge Domains and Journal Distribution

To better understand how research on ABFs is organized within the scientific literature, analyzing journal distribution and subject areas provides a perspective on where research outputs are concentrated, which can guide readers to key publication venues and highlight the domains driving advances in antioxidant biopolymeric films. Accordingly, Table 3 presents the ranking of the ten most relevant journals and subject areas. Over the ten-year study period, these journals accounted for 43.55% of the 953 published works, indicating a strong concentration of contributions within a limited number of specialized publication venues. Although Trends in Food Science and Technology exhibited the highest impact factor (IF = 15.4, 2024), the relevance of individual studies is not determined solely by journal metrics but by their influence on subsequent material development and technological adoption.

The distribution across subject areas shows that Agricultural and Biological Sciences (44.39%), Chemistry (35.57%), and Materials Science (33.16%) dominate the field, whereas Pharmacology, Toxicology, and Pharmaceutics represent only 4.72% of publications. This pattern demonstrates that antioxidant biopolymeric films are positioned at the interface between application-driven research and materials engineering, where polymer functionality is tailored to specific performance requirements rather than investigated as a purely biomedical material.

Previous analyses have emphasized the complexity of conventional plastic formulations, which may contain thousands of chemical additives with potential environmental and health implications [34]. Consequently, current research trends increasingly focus on polymer systems derived from renewable sources that allow property modulation through chemical structure and processing rather than through synthetic additive packages. In this context, biodegradable polymer families such as polylactic acid (PLA), bio-polystyrene, starch-based blends, polyhydroxyalkanoates (PHA), and bacterial cellulose have emerged as model matrices for designing functional packaging materials [35].

Together, these observations indicate that the field is evolving from material replacement strategies toward engineered polymer systems in which performance derives from the intrinsic characteristics of the macromolecular network and its interaction with incorporated active compounds.

## 4. Hot Research Topics

**Figure 4:**
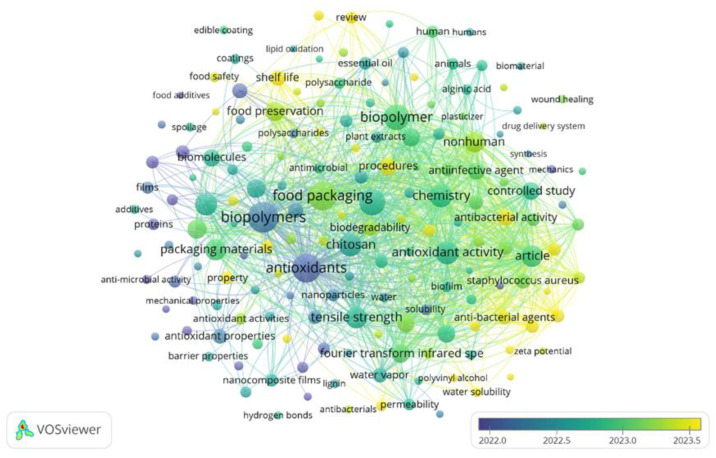
Keyword co-occurrence network (2015–2025) revealing the conceptual relationships linking materials composition, functional activity, and application performance in antioxidant biopolymeric films. Bibliometric data were retrieved from the Scopus database Data collection was performed on 17 September 2025. Keyword co-occurrence analysis was performed using VOSviewer (version 1.6.20) with a minimum occurrence threshold of 25 keywords.

**Figure 5:**
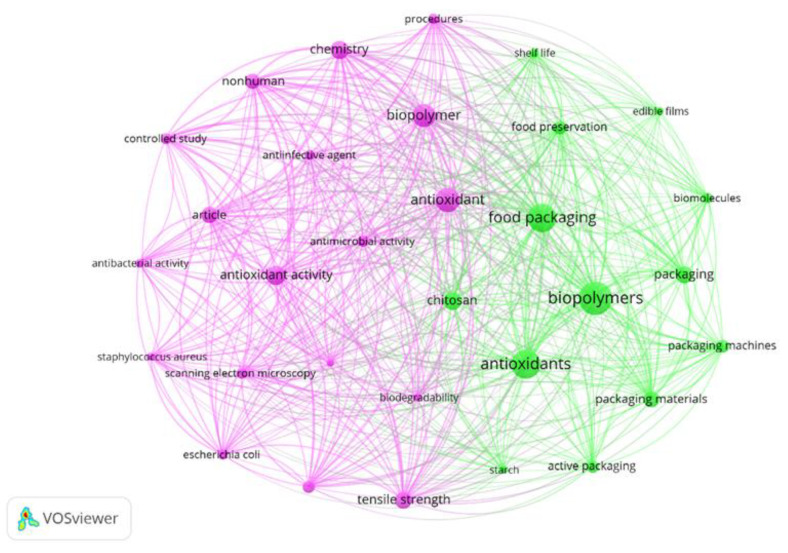
High-frequency keyword clustering (2015–2025) demonstrating the transition from biodegradable films to active functional polymer systems in antioxidant biopolymeric films. Bibliometric data were retrieved from the Scopus database between 2015 and 2025. Data collection was performed on 17 September 2025. The clustering map includes only high-frequency keywords (minimum occurrence ≥ 100) to highlight dominant research themes.

Keyword analysis enables identification of conceptual relationships governing the design and functional performance of ABFs. A total of 6906 keywords were extracted from the 953 analyzed articles, of which 169 appeared at least 25 times (Figure 4). When the minimum occurrence threshold increased to 100, 31 dominant descriptors remained (Figure 5), representing the core conceptual structure of the field. In these maps, node size is proportional to frequency of occurrence.

The temporal distribution of keywords shows a pronounced concentration between 2022 and 2023, coinciding with the period of greatest growth in publications and citations. Table 4 lists the descriptors with frequency above 200. As expected, given the search strategy, terms such as biopolymers (f = 437), antioxidants (f = 385), and food packaging (f = 379) are among the most frequent. The co-occurrence patterns associated with these terms demonstrate that research is no longer centered on biodegradable substitution but on functional performance. The shift from “biodegradable films” to “active packaging” indicates a transition toward second-generation biopolymers designed to interact chemically with packaged systems rather than merely isolate them.

Emerging descriptors such as food preservation (f = 174), antimicrobial activity (f = 156), and starch (f = 106) reveal increasing interest in multifunctional materials. Rather than acting as passive barriers, polymeric films are being engineered as controlled-release media in which antioxidant molecules modulate oxidation reactions through diffusion and interfacial interactions.

The clustering pattern also shows a growing association between mechanical descriptors and chemical functionality. The recurrent coupling of “tensile strength”, “chitosan”, and “antioxidant activity” reflects an effort to simultaneously regulate structural integrity and molecular transport, properties that are typically antagonistic in hydrophilic polymer networks [36,37]. This convergence indicates that current research focuses on balancing barrier performance, mechanical resistance, and release kinetics, consolidating ABFs as engineered polymer systems rather than environmentally motivated materials.

Barzan et al. [37] report innovations in food packaging production using extracts from Moringa oleifera leaves as a source of antioxidant compounds. These extracts enhanced antioxidant properties, reducing the lipid peroxidation process of beef proteins by 50% after 16 days of exposure under the study conditions. This system is considered promising and, most importantly, sustainable for industrial applications in food production.

It is important to note that Table 4 presents the most frequent indexed keywords, excluding generic database-assigned terms (e.g., “Article”, “Nonhuman”) to focus strictly on conceptual scientific variables. Additionally, semantically related terms (e.g., “antioxidant”, “antioxidants”, and “antioxidant activity”) were not merged in order to preserve the original granularity of the dataset and avoid subjective normalization, ensuring that the analysis reflects the actual distribution of descriptors in the literature.

The predominance of descriptors such as “biopolymer”, “antioxidant”, and “film” reflects the central focus of the field. However, it should be acknowledged that the prominence of certain descriptors partially reflects the search strategy employed; nevertheless, their co-occurrence patterns still provide meaningful insights into the thematic structure of the field. Additionally, the presence of terms related to specific matrices and bioactive compounds highlights emerging research directions.

The prevalence of these clusters, specifically focusing on polymer matrices, functional additives, and processing methods, provides the categorical framework for the following sections. Based on this bibliometric mapping, the subsequent chapters offer a thematic transition from quantitative trends to a descriptive analysis of the materials and design strategies that currently define the state of the art in ABFs.

Collectively, these keyword patterns further reinforce the proposed thesis by demonstrating that current research emphasis is no longer limited to biodegradability, but is increasingly focused on multifunctional performance, controlled release behavior, and structure–property optimization.

## 5. Materials and Design Strategies in Antioxidant Biopolymeric Films (ABFs)

### 5.1. Biopolymer Matrices

In line with the central thesis of this review, the following sections move beyond bibliometric observation to demonstrate how this conceptual shift is reflected in material selection and design strategies.

The mapping of high-frequency keyword clusters (Figure 4 and Figure 5) identifies a research landscape centered on the synergy between biopolymer matrices, such as chitosan and proteins, and the optimization of barrier and mechanical properties. Driven by these core trends, the design of ABFs focuses on tailoring the polymer structure to achieve controlled release and extended food shelf life. Consequently, the selection of the polymer matrix represents the primary design variable governing the performance of ABFs. Rather than acting solely as structural supports, these matrices control molecular mobility, oxygen permeability, and the release kinetics of incorporated active compounds. Consequently, antioxidant efficiency depends on the physicochemical compatibility between polymer chains and bioactive molecules rather than on polymer origin alone.

While chitosan stands out in the bibliometric dataset (Table 4), the broader literature highlights a diverse range of investigated biopolymers, including starch, cellulose, gelatin, kappa-carrageenan, alginates, and polyvinyl alcohol. Across the literature, polysaccharides and proteins dominate due to their high density of polar functional groups, which promotes intermolecular interaction with antioxidant compounds. However, the same interactions increase hydrophilicity and water sensitivity, explaining the widespread use of blended and composite matrices. Current material selection therefore prioritizes intermolecular compatibility and controlled diffusion over biodegradability as an isolated criterion [38,39,40,41,42,43,44].

Different polymer chemistries present distinct charge densities, hydrogen-bonding capacity, and chain flexibility, which directly influence additive dispersion and migration behavior. Protein-based matrices, such as gelatin, typically form networks characterized by high chain entanglement and specific secondary structures (α-helices and β-sheets). These structures often result in a more flexible amorphous network compared to some crystalline polysaccharides, which facilitates molecular mobility and favors diffusion-controlled release of bioactive compounds [45,46].

In contrast, polysaccharides often generate dense intermolecular networks stabilized by extensive hydrogen bonding. While this high degree of interaction provides an effective barrier against non-polar gases like oxygen (O_2_), their performance is highly sensitive to environmental conditions. Due to the abundance of hydroxyl and other polar groups, polysaccharides are inherently hydrophilic; thus, they tend to exhibit moderate to low barrier efficiency against water vapor, as moisture plasticizes the network and increases permeability [47,48,49]. Blends combining natural and synthetic polymers emerge as intermediate systems capable of decoupling permeability from mechanical resistance [38,43,50,51,52,53,54,55,56].

Overall, the performance of ABFs is governed by a triad consisting of polymer polarity, additive compatibility, and processing-induced microstructure. Hydrophilic matrices enhance antioxidant dispersion but increase permeability, while semi-crystalline structures improve barrier properties at the expense of release kinetics. Consequently, recent developments increasingly employ composite and multilayer architectures to balance diffusion processes and structural stability.

### 5.2. Processing and Film Formation Technologies

Processing conditions play a decisive role in determining the functional performance of ABFs because they regulate chain organization, free volume distribution, and transport pathways within the polymer network. The principal fabrication techniques include casting, coating, layer-by-layer assembly, and extrusion [57], each producing distinct microstructural arrangements that directly affect permeability and release kinetics.

Solution casting typically generates relatively homogeneous matrices with higher molecular mobility, which facilitates rapid antioxidant migration. In contrast, extrusion and multilayer assembly promote denser packing and interfacial structuring, improving barrier resistance while reducing diffusion rates. Consequently, film functionality is governed not only by composition but by the morphology established during processing [42,51,58].

Variations in formulation parameters, such as plasticizer concentration, solvent evaporation rate, mixing intensity, and layer deposition interval, modify intermolecular interactions and phase distribution. For example, multilayer systems prepared with alternating biopolymer compositions can create gradient diffusion pathways, whereas blended formulations processed by casting tend to produce single-phase transport behavior [38,40,41,42,51].

Both pre- and post-processing treatments further reinforce these structure–property relationships. High-pressure homogenization, applied prior to film formation, increases molecular interaction density and thermal stability by reducing structural heterogeneity [58], while solvent vapor annealing induces polymer rearrangement and partial crystallization through swelling-driven self-assembly [59]. These approaches demonstrate that fabrication is effectively a structural tuning step, enabling control over the balance between oxygen barrier properties and antioxidant release.

Overall, the wide range of processing strategies highlights that antioxidant performance in bio-based films derives primarily from processing-induced morphology rather than solely from chemical formulation. The incorporation of bioactive compounds therefore becomes effective only when compatible with the transport characteristics defined during film formation.

In practice, no single processing technique can be considered universally optimal. However, solution casting is generally the most suitable approach when high antioxidant activity is required due to its promotion of rapid diffusion and homogeneous dispersion of bioactive compounds, whereas extrusion and multilayer structures are preferable for applications prioritizing barrier performance and structural integrity. Therefore, the selection of the processing method should consider the target functionality, the nature of the polymer matrix, and the characteristics of the incorporated bioactive compounds.

### 5.3. Bioactive Functionalization

The incorporation of active compounds transforms packaging films into functional systems capable of interacting with the packaged medium. Compared to direct addition into food matrices, embedding bioactive compounds within polymeric materials enables controlled release, reduces required concentrations, and prolongs functional effectiveness. Consequently, antioxidant activity in packaging depends not only on chemical reactivity but also on migration behavior governed by the polymer network [19]. In this context, antioxidant migration can be interpreted as a diffusion-controlled transport phenomenon governed by polymer free volume, intermolecular interactions, and network morphology.

In general, polysaccharide-based matrices such as chitosan and starch are frequently associated with plant extracts rich in phenolic compounds, aiming to enhance antioxidant activity and control oxidative degradation. Protein-based matrices, including gelatin and whey proteins, are often combined with essential oils and low-molecular-weight antioxidants to improve film functionality and mechanical performance. These combinations highlight the need to balance barrier properties, antioxidant efficiency, and controlled release behavior.

A major challenge in designing these materials is balancing biodegradability, preservation efficiency, and consumer safety. This balance can be achieved by combining biopolymers with natural bioactive agents, such as grape seed and skin extracts, which provide antioxidant and antimicrobial properties while valorizing agro-industrial by-products within circular bioeconomy strategies [60].

From a materials perspective, bioactive compounds behave as low-molecular-weight species dispersed within a macromolecular matrix. Their performance depends on solubility, partition coefficient, and interaction with polymer functional groups rather than solely on intrinsic chemical activity. These compounds may originate from natural or synthetic sources and include polyphenols, capsaicinoids, peptides, and alkaloids. Extraction approaches vary according to raw material characteristics, ranging from conventional techniques to assisted and high-efficiency methods [19,26,61,62].

Two principal incorporation strategies are observed: direct dispersion of molecules and encapsulation in carrier structures. Free molecules provide rapid activity but are depleted quickly, whereas encapsulated systems allow sustained release through diffusion or gradual matrix degradation. Therefore, antioxidant efficiency is primarily determined by release kinetics and polymer-bioactive compatibility [38,40,51,54,63,64].

Beyond the physical entrapment of these species, the effectiveness of ABFs is increasingly attributed to synergistic interactions between the polymer matrix and the incorporated bioactive compounds. For instance, functional groups present in biopolymers, such as amino groups in chitosan and polyphenolic moieties in lignin, can interact with embedded antioxidants through mechanisms including hydrogen bonding and other secondary intermolecular interactions. These interactions may contribute to the stabilization of volatile active compounds and, in some cases, complement their function through the intrinsic radical scavenging capacity of the matrix components [61,65,66]. Such combined effects underpin the active behavior of the barrier system, in which the matrix and the additives act in a coordinated manner rather than as fully independent entities. Consequently, the antioxidant performance cannot be attributed solely to the additive, but rather to the coupled behavior of the system. This is consistent with a release-on-demand mechanism, whereby environmental triggers, such as moisture, may plasticize the polymer network and increase chain mobility, thereby facilitating controlled diffusion of active compounds in response to the degradation kinetics of the food system [67,68].

The addition of bioactive compounds alters the physicochemical behavior of the polymer network. Studies report modifications in thermal stability, enthalpy, and antioxidant performance following incorporation of compounds such as curcumin, capsaicin, silver nanoparticles, montmorillonite, and protein hydrolysates [38]. Other works demonstrate property changes after incorporation of plant extracts, essential oils, and nanostructured additives, which modify intermolecular interactions and transport pathways within the matrix [40,51,54,55,63,64]. Table 5 summarizes commonly reported bioactive compounds, their typical polymer matrices, and their functional roles.

Overall, bioactive functionalization should be understood as a diffusion-controlled stabilization mechanism in which the polymer matrix regulates the availability of reactive species. The effectiveness of ABFs therefore depends on interfacial compatibility, molecular mobility, and sustained migration rather than simply on the presence of antioxidant compounds [20,69].

**Table 5:** Overview of bioactive compounds, typical polymer matrices, and their functional roles in bio-based films.

Bioactive	Typical Matrix Type	Function	References
Essential oils (peppermint, oregano)	Polysaccharides (e.g., chitosan, alginate, starch, cellulose)	Antimicrobial, antioxidant	[36,44,70,71]
Plant extracts	Polysaccharides	Antimicrobial, antioxidant	[60,72]
Metal oxides (e.g., CuO nanoparticles)	Polysaccharides	Antimicrobial	[17,73]
Polyphenols (e.g., curcumin, quercetin)	Polysaccharides, proteins (e.g., gelatin, soy protein, collagen)	Antioxidant, antimicrobial	[38,62,74,75]
Fat-soluble vitamins (e.g., tocopherol), carotenoids	Polysaccharides	Antioxidant	[16,76]

## 6. Structure–Property Relationships and Characterization

Central to the performance of ABFs is the balance between barrier efficiency and controlled molecular transport: the material must be sufficiently dense to limit gas permeability, while maintaining adequate chain mobility to enable the regulated diffusion of antioxidant compounds. This transport process is not a secondary effect but a key functional mechanism of the system. Accordingly, characterization efforts should focus on understanding how structural features of the polymer matrix, such as free volume and segmental mobility, influence the diffusion pathways of the active species. If the interactions between the polymer and the antioxidant are excessively strong, the additive may become partially immobilized, thereby limiting its effective release despite a high overall loading. These considerations support the interpretation of ABFs as coupled transport systems, in which barrier properties and controlled release are intrinsically interdependent [20,69,77].

The functional performance of ABFs emerges from the relationship between microstructure and transport phenomena. Variations in composition and processing alter chain organization, which in turn controls permeability, solubility, and release behavior. For instance, Delgado et al. [78] demonstrated that plasticizer concentration primarily influences solubility, whereas film thickness strongly affects water vapor permeability, highlighting the dominant role of diffusion pathways over chemical composition alone.

Characterization techniques reported in the literature aim to establish correlations between molecular organization and mass transport behavior. Mechanical, thermal, and spectroscopic analyses consistently indicate that antioxidant activity depends on chain mobility, crystallinity, and intermolecular interactions. Highly ordered structures improve barrier properties but limit antioxidant diffusion, whereas amorphous networks favor migration at the expense of structural stability. Therefore, effective materials require balancing molecular mobility with cohesive integrity. These observations reinforce that ABFs should be interpreted as diffusion-controlled polymer systems rather than simply biodegradable materials containing antioxidants.

Experimental characterization typically combines morphological and physicochemical analyses. For example, Nowak et al. [38] evaluated nanoparticle dispersion, surface morphology (AFM and SEM), optical properties, thickness, moisture content, and solubility, followed by thermal analysis (DSC) and antioxidant activity measurements (FRAP and metal chelation). Such multiscale approaches allow linking nanoscale organization to macroscopic functional performance in food preservation applications.

Complementary techniques further clarify structure–property relationships, including contact angle measurements, mechanical testing, permeability assays, spectroscopic analyses (ATR-FTIR and NMR), crystallinity determination (XRD), thermal stability (TGA), and release kinetics evaluation. These methods collectively describe polymer–bioactive interactions and diffusion behavior within the matrix rather than isolated material properties [52,54,79,80].

Overall, characterization in this field functions as an integrative tool to understand ABFs as coupled transport systems in which barrier performance and molecular release are interdependent phenomena. This perspective aligns with the classical materials science paradigm in which processing, structure, and properties are interdependent variables governing material performance.

## 7. Application Fields and Technological Readiness

Antioxidant biopolymeric films have potential applications in pharmaceutical, cosmetic, and packaging systems; however, the predominance of food-related keywords (Table 4) indicates that food preservation serves as the primary validation platform for evaluating functional polymer performance. In this context, shelf-life extension results from controlled molecular migration rather than from simple physical protection [60].

Application studies consistently demonstrate that these materials primarily act by delaying lipid oxidation, whereas antimicrobial effects are secondary and dependent on release rate. Therefore, packaging efficiency depends on matching antioxidant diffusion kinetics with the oxidation rate of the stored product, confirming that these materials operate as time-dependent transport systems rather than passive barriers.

Biopolymeric materials are typically applied as free-standing films or edible coatings for sustainable food packaging systems [71], both acting as diffusion-regulating layers. For example, multilayer coatings applied to Atlantic salmon showed oxidation delay associated with sustained release behavior [38], while coatings applied to cooked ham preserved color stability and reduced oxidative degradation [64]. Similar effects were observed in fresh fruits: antimicrobial films applied to figs [54] and sealing films used in refrigerated apples [58] maintained quality by regulating oxygen permeability and antioxidant availability rather than by complete microbial inhibition.

These results demonstrate that application performance is governed by synchronization between transport properties of the polymer matrix and degradation kinetics of the packaged product. Consequently, the technological readiness of ABFs depends on the ability to tailor release profiles to specific oxidation mechanisms instead of relying solely on the presence of active compounds.

## 8. Research Gaps and Future Polymer Engineering Directions

Current research on ABFs is predominantly oriented toward food preservation; however, the main limitation of biodegradable polymers remains their insufficient mechanical resistance and barrier performance [19,79,81]. Recent studies therefore increasingly employ polymer blending strategies to tailor physicochemical behavior, indicating a transition from single-component materials to engineered multiphase systems. Beyond food applications, the incorporation of bioactive compounds also opens opportunities in biomedical materials, where controlled release and biocompatibility become critical design parameters [23,53].

A central challenge lies in simultaneously optimizing mechanical integrity and functional activity. Reinforcement approaches demonstrate that structural modifiers can significantly improve performance: incorporation of spider silk enhances tensile strength in chitosan–starch matrices [82], while protein-rich agricultural residues improve both mechanical properties and antioxidant activity [83]. These findings highlight the importance of identifying reinforcing phases capable of modifying intermolecular interactions without compromising transport behavior.

Although plant-derived antioxidants are widely explored [42,51,54], the diversity of available bioactive sources remains underutilized. Emerging studies indicate the potential of alternative biological metabolites, such as algae-derived compounds [84] and endophytic fungal metabolites [85], suggesting that future materials may rely on broader biochemical diversity to tune release profiles and functional stability. These materials therefore represent a promising strategy for integrating circular bioeconomy principles with sustainable food packaging technologies.

Another critical research gap concerns the relationship between laboratory preparation and industrial manufacturing. The performance of ABFs depends strongly on processing variables and their interaction with incorporated molecules [26,58]. Therefore, future work should prioritize establishing processing–structure–property relationships and scalable fabrication strategies capable of ensuring reproducibility and consistent release kinetics. Addressing these aspects is essential to bridge the gap between experimental materials and commercially viable polymer systems.

Despite advances in ABF development, a significant challenge remains in the precise engineering of polymer–additive synergism. Future studies should move beyond simple blending toward designing matrices in which antioxidant release is optimally synchronized with the oxidative kinetics of the food product. The ability to tailor these release profiles by manipulating the molecular interactions between bioactive compounds and biopolymer chains will be essential to bridge the gap between laboratory-scale films and commercially viable, high-performance active packaging systems [20,67,86].

## 9. Conclusions

The bibliometric mapping indicates that research on antioxidant biopolymeric films has progressed from environmentally motivated material substitution to performance-driven polymer engineering. The significant growth in publications from 2015 to 2025 reflects a global shift toward sustainable food packaging, with India, China, Brazil, Iran, and Spain emerging as the leading contributors. This geographical distribution is driven by a combination of vast agricultural biomass availability and stringent environmental policies. Key research hubs, such as Kyung Hee University, University of São Paulo, and Tabriz University of Medical Sciences, have been instrumental in bridging Agricultural and Biological Sciences with Materials Science. The synergy between these fields has shifted the focus from simple material substitution to the development of high-performance Antioxidant Biopolymeric Films (ABFs) tailored for the food industry.

These materials no longer function as passive barriers but as coupled transport–reaction systems, in which oxidation control depends on the balance between oxygen permeability and antioxidant migration. Polysaccharide and protein matrices dominate current developments due to their chemical compatibility with bioactive compounds; however, their inherent hydrophilicity introduces a trade-off between barrier resistance and controlled release. As a result, recent studies increasingly employ blends, composites, and multilayer architectures to decouple mechanical stability from diffusion behavior. Processing conditions play a decisive role in this balance, as microstructure governs molecular mobility within the polymer network.

Evidence across the literature shows that antioxidant effectiveness is determined primarily by polymer–additive interactions and matrix morphology rather than additive concentration alone. The next stage of the field requires predictive processing–structure–property relationships. Consequently, the development of ABFs should be approached as a polymer engineering problem in which functional performance emerges from the coupling between chemical composition, processing conditions, and transport phenomena.

Overall, the field is evolving toward engineered functional materials tailored to match the degradation kinetics of specific systems. Advances will rely on scalable fabrication strategies, improved compatibility between matrices and active compounds, and the design of multifunctional architectures. From this perspective, ABFs should be understood as functional bio-based polymer systems designed for sustainable food packaging, whose performance emerges from controlled mass transport and polymer–bioactive interactions. This conceptual shift indicates that future advances will arise from polymer engineering strategies rather than from the discovery of new additives alone. This framework repositions ABFs within polymer science as functional transport materials whose design must be guided by reaction–diffusion coupling rather than additive selection.

## Figures and Tables

**Table 1:** Scopus search results illustrating the growth and convergence of research domains related to antioxidant biopolymeric films (all years vs. 2015–2025).

Keywords	Total Number of Papers ^a^	Total Number of Papers in the Last 10 Years ^a^
biopolymer*	62,515	38,472
antioxidant*	595,177	398,384
film*	1,290,826	537,813
biopolymer* AND antioxidant*	2886	2660
biopolymer* AND film*	7774	5961
antioxidant* AND film*	8941	7687
biopolymer* AND antioxidant* AND film*	976	953

* Truncation symbol. ^a^ Data retrieved from the Scopus database illustrating the evolution from individual descriptors to the final integrated query TITLE-ABS-KEY (biopolymer* AND antioxidant* AND film*), restricted to research and review articles published in English. Data collection date: 17 September 2025. The time filter (2015–2025) was applied to evaluate recent research trends; values without restriction correspond to the entire database coverage.

**Table 2:** Major research contributors and collaborative hubs in antioxidant biopolymeric films research based on publication output (2015–2025).

Rank	Name	Number of Publications ^a^	Percentage	Citations	H-Index
Countries					
1	India	185	19.41	5108	41
2	China	142	14.90	5960	43
3	Brazil	123	12.91	3469	34
4	Iran	101	10.60	5147	38
5	Spain	71	7.45	3787	31
6	South Korea	63	6.61	2906	29
7	Turkey	59	6.19	1362	19
8	United States	51	5.35	2839	25
9	Poland	48	5.04	1925	24
10	Italy	37	3.88	1335	21
Institutions					
1	Kyung Hee University	35	3.67	1897	20
2	Universidade de São Paulo	24	2.52	853	13
2	Tabriz University of Medical Sciences	24	2.52	1240	14
4	Uniwersytet Rolniczy im. Hugona Kołłątaja w Krakowie	23	2.41	1228	16
5	Universidade Estadual de Campinas	21	2.20	935	14
6	Faculty of Nutrition and Food Sciences	20	2.10	1169	12
7	Hainan University	17	1.78	550	9
7	University of Nizwa	17	1.78	209	6
7	University of Petroleum and Energy Studies	17	1.78	209	6
10	Consejo Nacional de Investigaciones Científicas y Técnicas	16	1.68	393	9
10	Consejo Superior de Investigaciones Científicas	16	1.68	547	11
10	Lovely Professional University	16	1.68	377	8
Authors					
1	Jong-Whan Rhim	28	2.94	1806	19
2	Ewelina Jamróz	20	2.10	1021	15
3	Swarup Roy	18	1.89	1091	12
4	Ahmed Sulaiman Al-Harrasi	17	1.78	209	6
5	Wanli Zhang	16	1.68	604	10
5	Saurabh C. Bhatia	16	1.68	181	5
7	Milad Tavassoli	15	1.57	692	8
7	Ali Ehsani	15	1.57	632	10
9	Esra Koca	14	1.47	78	4
9	Levent Yurdaer Aydemir	14	1.47	78	4

^a^ Data retrieved from the Scopus database. Data collection date: 17 September 2025. Country and institution attribution are based on author affiliations indexed in Scopus. The h-index values refer only to the dataset retrieved in this study and do not represent the authors’ overall bibliometric indicators.

**Table 3:** Distribution of publications across journals and scientific domains highlighting the interdisciplinary positioning of antioxidant biopolymeric films research (2015–2025).

Rank	Name	Number of Publications ^a^	Percentage	IF (2024) ^b^
Journals				
1	*International Journal of Biological Macromolecules*	153	16.05	8.5
2	*Polymers*	55	5.77	4.9
3	*Food Hydrocolloids*	43	4.51	12.4
4	*Food Chemistry*	33	3.46	9.8
5	*Food Packaging and Shelf Life*	31	3.25	10.6
6	*Trends in Food Science and Technology*	25	2.62	15.4
7	*Carbohydrate Polymers*	23	2.41	12.5
8	*Food and Bioprocess Technology*	18	1.89	5.8
9	*Journal of Food Measurement and Characterization*	17	1.78	3.3
10	*Foods*	17	1.78	5.1
Subject Area				
1	Agricultural and Biological Sciences	423	44.39	
2	Chemistry	339	35.57	
3	Materials Science	316	33.16	
4	Biochemistry, Genetics and Molecular Biology	281	29.49	
5	Chemical Engineering	184	19.31	
6	Engineering	138	14.48	
7	Physics and Astronomy	62	6.51	
8	Medicine	52	5.46	
9	Environmental Science	50	5.25	
10	Pharmacology, Toxicology and Pharmaceutics	45	4.72	

^a^ Data retrieved from the Scopus database. Data collection date: 17 September 2025. Subject areas correspond to Scopus classification categories. Journal impact factors refer to the most recent values available at the time of analysis. ^b^ IF = Impact Factor.

**Table 4:** Dominant research descriptors revealing functional design priorities in antioxidant biopolymeric films (frequency > 200, 2015–2025).

Rank	Keywords	Frequency ^a^
1	Biopolymers	437
2	Antioxidants	385
3	Food Packaging	379
4	Antioxidant	317
5	Biopolymer	305
6	Antioxidant Activity	254
7	Chitosan	242
8	Chemistry	239
9	Packaging	228
10	Tensile Strength	223
11	Packaging Materials	212

^a^ Data retrieved from the Scopus database. Data collection date: 17 September 2025. Keyword frequency was calculated from author keywords indexed in Scopus. Only terms with occurrence greater than 200 were included.

## Data Availability

No new data were created or analyzed in this study. Data sharing is not applicable to this article.

## Abbreviations

The following abbreviations are used in this manuscript:

ABFs	Antioxidant biopolymeric films
ABTS	2,2′-azino-bis (3-ethylbenzothiazoline-6-sulfonic acid)
AFM	Atomic force microscopy
AgNPs	Silver nanoparticles
ATR	Attenuated total reflection
DPPH	2,2-diphenyl-1-picrylhydrazyl
DSC	Differential scanning calorimetry
f	Frequency
FRAP	Ferric reducing antioxidant power
GD	Degree of deacetylation
HPMC	Hydroxypropyl methyl cellulose
IF	Impact factor
FTIR	Fourier transform infrared spectroscopy
NMR	Nuclear magnetic resonance
PHA	Polyhydroxyalkanoate
PLA	Polylactic acid
POCs	Persistent organic compounds
PVA	Polyvinyl alcohol
SEM	Scanning electron microscopy
SDGs	Sustainable development goals
TGA	Thermogravimetric analysis
XRD	X-ray diffraction

## References

[1] Saravanan A., Kumar P.S., Hemavathy R.V., Jeevanantham S., Harikumar P., Priyanka G., Devakirubai D.R.A. (2022). A Comprehensive Review on Sources, Analysis and Toxicity of Environmental Pollutants and Its Removal Methods from Water Environment. Sci. Total Environ..

[2] Arora-Jonsson S. (2023). The Sustainable Development Goals: A Universalist Promise for the Future. Futures.

[3] Sorooshian S. (2024). The Sustainable Development Goals of the United Nations: A Comparative Midterm Research Review. J. Clean. Prod..

[4] Mishra A., Kumari M., Kumar R., Iqbal K., Thakur I.S. (2022). Persistent Organic Pollutants in the Environment: Risk Assessment, Hazards, and Mitigation Strategies. Bioresour. Technol. Rep..

[5] Moulatlet G.M., Yacelga N., Rico A., Mora A., Hauser-Davis R.A., Cabrera M., Capparelli M.V. (2023). A Systematic Review on Metal Contamination Due to Mining Activities in the Amazon Basin and Associated Environmental Hazards. Chemosphere.

[6] Déciga-Alcaraz A., Tlazolteotl Gómez de León C., Morales Montor J., Poblano-Bata J., Martínez-Domínguez Y.M., Palacios-Arreola M.I., Amador-Muñoz O., Rodríguez-Ibarra C., Vázquez-Zapién G.J., Mata-Miranda M.M. (2023). Effects of Solvent Extracted Organic Matter from Outdoor Air Pollution on Human Type II Pneumocytes: Molecular and Proteomic Analysis. Environ. Pollut..

[7] Dutta S., Adhikary S., Bhattacharya S., Roy D., Chatterjee S., Chakraborty A., Banerjee D., Ganguly A., Nanda S., Rajak P. (2024). Contamination of Textile Dyes in Aquatic Environment: Adverse Impacts on Aquatic Ecosystem and Human Health, and Its Management Using Bioremediation. J. Environ. Manag..

[8] Zhou W., Li M., Achal V. (2025). A Comprehensive Review on Environmental and Human Health Impacts of Chemical Pesticide Usage. Emerg. Contam..

[9] Huang Y., Li Z. (2024). Assessing Pesticides in the Atmosphere: A Global Study on Pollution, Human Health Effects, Monitoring Network and Regulatory Performance. Environ. Int..

[10] Barathe P., Kaur K., Reddy S., Shriram V., Kumar V. (2024). Antibiotic Pollution and Associated Antimicrobial Resistance in the Environment. J. Hazard. Mater. Lett..

[11] Ziliotto M., Chies J.A.B., Ellwanger J.H. (2024). Toxicogenomics of Persistent Organic Pollutants: Potential Impacts on Biodiversity and Infectious Diseases. Anthropocene.

[12] Kushwaha M., Shankar S., Goel D., Singh S., Rahul J., Rachna K., Singh J. (2024). Microplastics Pollution in the Marine Environment: A Review of Sources, Impacts and Mitigation. Mar. Pollut. Bull..

[13] Pegado T.d.S.e.S., Schmid K., Winemiller K.O., Chelazzi D., Cincinelli A., Dei L., Giarrizzo T. (2018). First Evidence of Microplastic Ingestion by Fishes from the Amazon River Estuary. Mar. Pollut. Bull..

[14] Rojas R.R., Arango-Mora C., Nolorbe-Payahua C., Medina M., Vasquez M., Flores J., Murayari F., Vásquez C., Almeida V.D., Ramos W. (2023). Microplastic Occurrence in Fish Species from the Iquitos Region in Peru, Western Amazonia. Acta Amaz..

[15] (2018). The Future of Plastic. Nat. Commun..

[16] de Oliveira Filho J.G., Bertolo M.R.V., Fernandes S.S., Lemes A.C., da Cruz Silva G., Junior S.B., de Azeredo H.M.C., Mattoso L.H.C., Egea M.B. (2024). Intelligent and Active Biodegradable Biopolymeric Films Containing Carotenoids. Food Chem..

[17] Saravanakumar K., Sathiyaseelan A., Mariadoss A.V.A., Xiaowen H., Wang M.-H. (2020). Physical and Bioactivities of Biopolymeric Films Incorporated with Cellulose, Sodium Alginate and Copper Oxide Nanoparticles for Food Packaging Application. Int. J. Biol. Macromol..

[18] Dehghani S., Hosseini S.V., Regenstein J.M. (2018). Edible Films and Coatings in Seafood Preservation: A Review. Food Chem..

[19] Revutskaya N., Polishchuk E., Kozyrev I., Fedulova L., Krylova V., Pchelkina V., Gustova T., Vasilevskaya E., Karabanov S., Kibitkina A. (2024). Application of Natural Functional Additives for Improving Bioactivity and Structure of Biopolymer-Based Films for Food Packaging: A Review. Polymers.

[20] Almasi H., Jahanbakhsh Oskouie M., Saleh A. (2021). A Review on Techniques Utilized for Design of Controlled Release Food Active Packaging. Crit. Rev. Food Sci. Nutr..

[21] Frederiksen K., Guy R.H., Petersson K. (2016). The Potential of Polymeric Film-Forming Systems as Sustained Delivery Platforms for Topical Drugs. Expert Opin. Drug Deliv..

[22] Sahraee S., Milani J.M., Regenstein J.M., Kafil H.S. (2019). Protection of Foods against Oxidative Deterioration Using Edible Films and Coatings: A Review. Food Biosci..

[23] Falcão L.D.S., Coelho D.B., Veggi P.C., Campelo P.H., Albuquerque P.M., de Moraes M.A. (2022). Starch as a Matrix for Incorporation and Release of Bioactive Compounds: Fundamentals and Applications. Polymers.

[24] Benbettaïeb N., Debeaufort F., Karbowiak T. (2019). Bioactive Edible Films for Food Applications: Mechanisms of Antimicrobial and Antioxidant Activity. Crit. Rev. Food Sci. Nutr..

[25] Lai W.-F. (2021). Design of Polymeric Films for Antioxidant Active Food Packaging. Int. J. Mol. Sci..

[26] Jamróz E., Kopel P. (2020). Polysaccharide and Protein Films with Antimicrobial/Antioxidant Activity in the Food Industry: A Review. Polymers.

[27] Magadán-Díaz M., Rivas-García J.I. (2022). Publishing Industry: A Bibliometric Analysis of the Scientific Production Indexed in Scopus. Publ. Res. Q..

[28] Wang J., Chi Y., Yang B., Zhang Q., Wang D., He X., Li H. (2022). The Application of Biomaterials in Osteogenesis: A Bibliometric and Visualized Analysis. Front. Bioeng. Biotechnol..

[29] Rusydiana A.S. (2021). Bibliometric Analysis of Journals, Authors, and Topics Related to COVID-19 and Islamic Finance Listed in the Dimensions Database by Biblioshiny. Sci. Ed..

[30] Donthu N., Kumar S., Mukherjee D., Pandey N., Lim W.M. (2021). How to Conduct a Bibliometric Analysis: An Overview and Guidelines. J. Bus. Res..

[31] Zhu S., Liu Y., Gu Z., Zhao Y. (2021). A Bibliometric Analysis of *Advanced Healthcare Materials*: Research Trends of Biomaterials in Healthcare Application. Adv. Healthc. Mater..

[32] Muktiarni M., Widiaty I., Widaningsih L., Yulia C., Kitaw Dejene B. (2025). Advances in Thermoplastic Starch (TPS) Research: Bibliometric Analysis of Its Contribution to Sustainable Packaging and Environmental Sustainability. J. Adv. Res. Fluid Mech. Therm. Sci..

[33] Tavassoli-Kafrani E., Shekarchizadeh H., Masoudpour-Behabadi M. (2016). Development of Edible Films and Coatings from Alginates and Carrageenans. Carbohydr. Polym..

[34] Walker T.R., Fequet L. (2023). Current Trends of Unsustainable Plastic Production and Micro(Nano)Plastic Pollution. TrAC Trends Anal. Chem..

[35] Singh N., Ogunseitan O.A., Wong M.H., Tang Y. (2022). Sustainable Materials Alternative to Petrochemical Plastics Pollution: A Review Analysis. Sustain. Horiz..

[36] Guo Q., Du G., Jia H., Fan Q., Wang Z., Gao Z., Yue T., Yuan Y. (2021). Essential Oils Encapsulated by Biopolymers as Antimicrobials in Fruits and Vegetables: A Review. Food Biosci..

[37] Barzan G., Sacco A., Giovannozzi A.M., Portesi C., Schiavone C., Salafranca J., Wrona M., Nerín C., Rossi A.M. (2024). Development of Innovative Antioxidant Food Packaging Systems Based on Natural Extracts from Food Industry Waste and Moringa Oleifera Leaves. Food Chem..

[38] Nowak N., Tkaczewska J., Grzebieniarz W., Juszczak L., Mazur T., Szuwarzyński M., Guzik P., Jamróz E. (2024). Active and Intelligent Four-Layer Films Based on Chitosan, Gelatin, Furcellaran and Active Ingredients—Preparation, Characterisation and Application on Salmon. Food Bioprocess Technol..

[39] Jiang T., James R., Kumbar S.G., Laurencin C.T., Kumbar S.G., Laurencin C.T., Deng M. (2014). Chitosan as a Biomaterial. Natural and Synthetic Biomedical Polymers.

[40] de Paiva C.S., Batista F.G., Silva D.W., Scatolino M.V., de Medeiros D.T., Mascarenhas A.R.P., Lago R.C.D., Setter C., Borges I.O., Tonoli G.H.D. (2024). Andiroba Oil (Carapa Guianensis Aubletet) as a Functionalizing Agent for Titica Vine (Heteropsis Flexuosa) Nanofibril Films: Biodegradable Products from Species Native to the Amazon Region. Sustainability.

[41] Bajer D. (2024). Eco-Friendly, Biodegradable Starch-Based Packaging Materials with Antioxidant Features. Polymers.

[42] Bhatia S., Abbas Shah Y., Al-Harrasi A., Jawad M., Koca E., Aydemir L.Y. (2024). Enhancing Tensile Strength, Thermal Stability, and Antioxidant Characteristics of Transparent Kappa Carrageenan Films Using Grapefruit Essential Oil for Food Packaging Applications. ACS Omega.

[43] Salman H.H., Hussein A.A. (2024). Fabrication and Comperization Study the Effect of Molecular Weight for Chitosan Blended with Polyvinyl Alcohol for Food Packaging Application. Salud Cienc. Tecnol.—Ser. Conf..

[44] Rahman S., Batsh C., Gurumayam S., Borah J.C., Chowdhury D. (2024). Sodium Alginate-Nanocellulose-Based Active Composite Film for Edible Oils Packaging Applications. Mater. Adv..

[45] Pommet M., Redl A., Morel M.-H., Guilbert S. (2003). Study of Wheat Gluten Plasticization with Fatty Acids. Polymer.

[46] Fennema O.R., Damodaran S., Parkin K.L. (2017). Fennema’s Food Chemistry.

[47] Pavlath A.E., Orts W. (2009). Edible Films and Coatings: Why, What, and How?. Edible Films and Coatings for Food Applications.

[48] Gennadios A., Weller C.L., Gooding C.H. (1994). Measurement Errors in Water Vapor Permeability of Highly Permeable, Hydrophilic Edible Films. J. Food Eng..

[49] Banker G.S. (1966). Film Coating Theory and Practice. J. Pharm. Sci..

[50] Rigueto C.V.T., Rosseto M., Loss R.A., Richards N.S.P.D.S., Dettmer A., Pizzutti I.R. (2023). Gelatin-Based Polymeric Films for Applications in Food Packaging: An Overview of Advances, Challenges, and Perspectives. Ciência Rural.

[51] Murugan G., Nilsuwan K., Prodpran T., Ponnusamy A., Rhim J.-W., Kim J.T., Benjakul S. (2024). Active Fish Gelatin/Chitosan Blend Film Incorporated with Guava Leaf Powder Carbon Dots: Properties, Release and Antioxidant Activity. Gels.

[52] Croisier F., Jérôme C. (2013). Chitosan-Based Biomaterials for Tissue Engineering. Eur. Polym. J..

[53] Tincu (Iurciuc) C.E., Daraba O.M., Jérôme C., Popa M., Ochiuz L. (2024). Albumin-Based Hydrogel Films Covalently Cross-Linked with Oxidized Gellan with Encapsulated Curcumin for Biomedical Applications. Polymers.

[54] Kaur N., Somasundram C., Razali Z., Mourad A.-H.I., Hamed F., Ahmed Z.F.R. (2024). Aloe Vera/Chitosan-Based Edible Film with Enhanced Antioxidant, Antimicrobial, Thermal, and Barrier Properties for Sustainable Food Preservation. Polymers.

[55] Balciunaitiene A., Januskevice V., Saunoriute S., Raubyte U., Viskelis J., Memvanga P.B., Viskelis P. (2024). Antimicrobial Antioxidant Polymer Films with Green Silver Nanoparticles from Symphyti Radix. Polymers.

[56] Rochas C., Rinaudo M. (1984). Mechanism of Gel Formation in Κ-carrageenan. Biopolymers.

[57] Silva E.G.S., Cardoso S., Bettencourt A.F., Ribeiro I.A.C. (2023). Latest Trends in Sustainable Polymeric Food Packaging Films. Foods.

[58] Li F., Zhang F., Chen R., Ma Z., Wu H., Zhang Z., Yin S., Zhou M. (2023). Effects of High-Pressure Homogenization Treatment on the Development of Antioxidant Zanthoxylum Bungeanum Leaf Powder Films for Preservation of Fresh-Cut Apple. Foods.

[59] Băbuțan M., Botiz I. (2024). Morphological Characteristics of Biopolymer Thin Films Swollen-Rich in Solvent Vapors. Biomimetics.

[60] Ivanov Y., Godjevargova T. (2024). Antimicrobial Polymer Films with Grape Seed and Skin Extracts for Food Packaging. Microorganisms.

[61] Charles A.P.R., Rajasekaran B., Awasti N., Choudhary P., Khanashyam A.C., Majumder K., Wu Y., Pandiselvam R., Jin T.Z. (2025). Emerging Chitosan Systems Incorporated with Polyphenols: Their Applications in Intelligent Packaging, Active Packaging, and Nutraceutical Systems—A Comprehensive Review. Int. J. Biol. Macromol..

[62] Shafi Z., Singh R., Sidiqi U.S., Bashir B., Rasool S., Dash K.K., Zahoor I., Ahmed I., Nagaraja S.K., Dar A.H. (2025). Quercetin Infused Starch Matrix as a Sustainable Approach to Smart Packaging: A Comprehensive Review. Int. J. Biol. Macromol..

[63] Karabagias V.K., Giannakas A.E., Andritsos N.D., Leontiou A.A., Moschovas D., Karydis-Messinis A., Avgeropoulos A., Zafeiropoulos N.E., Proestos C., Salmas C.E. (2024). Shelf Life of Minced Pork in Vacuum-Adsorbed Carvacrol@Natural Zeolite Nanohybrids and Poly-Lactic Acid/Triethyl Citrate/Carvacrol@Natural Zeolite Self-Healable Active Packaging Films. Antioxidants.

[64] de Sousa Cândido G., Silva M.S., Zeneratto N.J., Piccoli R.H., Nunes Carvalho E.E., de Oliveira J.E. (2025). Hybrid Nanoclay/Clove Essential Oil in Cellulose Acetate Bionanocomposites for Cooked Ham Active Packaging. ACS Appl. Nano Mater..

[65] Amorati R., Valgimigli L. (2012). Modulation of the Antioxidant Activity of Phenols by Non-Covalent Interactions. Org. Biomol. Chem..

[66] Liu J., Liu S., Wu Q., Gu Y., Kan J., Jin C. (2017). Effect of Protocatechuic Acid Incorporation on the Physical, Mechanical, Structural and Antioxidant Properties of Chitosan Film. Food Hydrocoll..

[67] Kuai L., Liu F., Chiou B.-S., Avena-Bustillos R.J., McHugh T.H., Zhong F. (2021). Controlled Release of Antioxidants from Active Food Packaging: A Review. Food Hydrocoll..

[68] Mastromatteo M., Mastromatteo M., Conte A., Del Nobile M.A. (2010). Advances in Controlled Release Devices for Food Packaging Applications. Trends Food Sci. Technol..

[69] Duda J.L. (1985). Molecular Diffusion in Polymeric Systems. Pure Appl. Chem..

[70] Tian X., Huo X., Li X., Wang D., Lu J., Ren X., Kong Q. (2025). Characterization of the Sodium Alginate/Essential Oil Emulsion Film and Its Efficacy in Controlling Postharvest Penicillium Expansum Disease in Cherry Tomatoes. Int. J. Biol. Macromol..

[71] Cesca R.S., Fonseca G.G., Paz M.F.D., Cortez-Vega W.R. (2024). Advances and Perspectives on the Application of Essential Oils in Food Packaging Films, Coatings, and Nanoencapsulated Materials. Bragantia.

[72] Alves R.D.N., Grisi C.V.B., de Araújo R.N., Ferreira R.d.S.B., Pereira E.M., Cavalcanti M.T., da Silva W.P., Gonçalves M.C. (2025). Development and Characterization of Pink Pepper Extract Incorporated Chitosan, Guar Gum, Gelatin, and Palm Mucilage Based Active Film for Sustainable Food Packaging Applications. Int. J. Biol. Macromol..

[73] Kalia A., Kaur M., Shami A., Jawandha S.K., Alghuthaymi M.A., Thakur A., Abd-Elsalam K.A. (2021). Nettle-Leaf Extract Derived ZnO/CuO Nanoparticle-Biopolymer-Based Antioxidant and Antimicrobial Nanocomposite Packaging Films and Their Impact on Extending the Post-Harvest Shelf Life of Guava Fruit. Biomolecules.

[74] Ranade T., Sati A., Pratap A., Mali S.N. (2025). Curcumin-Integrated Biopolymer Films for Active Packaging: Current Trends and Future Directions. Chem. Pap..

[75] Ezati P., Rhim J.-W. (2021). Fabrication of Quercetin-Loaded Biopolymer Films as Functional Packaging Materials. ACS Appl. Polym. Mater..

[76] Pérez-Córdoba L.J., Norton I.T., Batchelor H.K., Gkatzionis K., Spyropoulos F., Sobral P.J.A. (2018). Physico-Chemical, Antimicrobial and Antioxidant Properties of Gelatin-Chitosan Based Films Loaded with Nanoemulsions Encapsulating Active Compounds. Food Hydrocoll..

[77] Peppas N.A., Sahlin J.J. (1989). A Simple Equation for the Description of Solute Release. III. Coupling of Diffusion and Relaxation. Int. J. Pharm..

[78] Delgado J.F., Peltzer M.A., Wagner J.R., Salvay A.G. (2018). Hydration and Water Vapour Transport Properties in Yeast Biomass Based Films: A Study of Plasticizer Content and Thickness Effects. Eur. Polym. J..

[79] Luo Q., Hossen M.A., Zeng Y., Dai J., Li S., Qin W., Liu Y. (2022). Gelatin-Based Composite Films and Their Application in Food Packaging: A Review. J. Food Eng..

[80] Rinaudo M., Milas M. (2000). Gellan Gum, a Bacterial Gelling Polymer. Novel Macromolecules in Food Systems.

[81] Pfaendner R. (2022). Restabilization—30 Years of Research for Quality Improvement of Recycled Plastics Review. Polym. Degrad. Stab..

[82] Kedir W.M., Geletu A.K., Weldegirum G.S. (2024). Spider Web-Reinforced Chitosan/Starch Biopolymer for Active Biodegradable Food Packaging. Appl. Food Res..

[83] Nie X., Shi H., Wang F., You C., Zhang D., Xiao Z., Li X. (2024). Biodegradable Chitosan-Based Biofilms Incorporated with Camellia Oleifera Residue Protein for Food Packaging. Food Hydrocoll..

[84] Esim N., Dawar P., Arslan N.P., Orak T., Doymus M., Azad F., Ortucu S., Albayrak S., Taskin M. (2024). Natural Metabolites with Antioxidant Activity from Micro-and Macro-Algae. Food Biosci..

[85] Asomadu R.O., Ezeorba T.P.C., Ezike T.C., Uzoechina J.O. (2024). Exploring the Antioxidant Potential of Endophytic Fungi: A Review on Methods for Extraction and Quantification of Total Antioxidant Capacity (TAC). 3 Biotech.

[86] de Jonge C.R.H.I. (1983). Synergism of Antioxidants. Pure Appl. Chem..

